# Knowledge translation within a population health study: how do you do it?

**DOI:** 10.1186/1748-5908-8-54

**Published:** 2013-05-21

**Authors:** Alison Kitson, Kathryn Powell, Elizabeth Hoon, Jonathan Newbury, Anne Wilson, Justin Beilby

**Affiliations:** 1 School of Nursing, The University of Adelaide, Adelaide 5005, Australia; 2 School of Population Health, The University of Adelaide, Adelaide 5005, Australia; 3 School of Medicine, Flinders University, Sturt Road Bedford Park, Adelaide, Australia; 4 Health Sciences, The University of Adelaide, Adelaide 5005, Australia

**Keywords:** Knowledge translation, Population health, Engaged scholarship, co-KT Framework, Health system redesign

## Abstract

**Background:**

Despite the considerable and growing body of knowledge translation (KT) literature, there are few methodologies sufficiently detailed to guide an integrated KT research approach for a population health study. This paper argues for a clearly articulated collaborative KT approach to be embedded within the research design from the outset.

**Discussion:**

Population health studies are complex in their own right, and strategies to engage the local community in adopting new interventions are often fraught with considerable challenges. In order to maximise the impact of population health research, more explicit KT strategies need to be developed from the outset. We present four propositions, arising from our work in developing a KT framework for a population health study. These cover the need for an explicit theory-informed conceptual framework; formalizing collaborative approaches within the design; making explicit the roles of both the stakeholders and the researchers; and clarifying what counts as evidence. From our deliberations on these propositions, our own co-creating (co-KT) Framework emerged in which KT is defined as both a theoretical and practical framework for actioning the intent of researchers and communities to co-create, refine, implement and evaluate the impact of new knowledge that is sensitive to the context (values, norms and tacit knowledge) where it is generated and used. The co-KT Framework has five steps. These include initial contact and framing the issue; refining and testing knowledge; interpreting, contextualising and adapting knowledge to the local context; implementing and evaluating; and finally, the embedding and translating of new knowledge into practice.

**Summary:**

Although descriptions of how to incorporate KT into research designs are increasing, current theoretical and operational frameworks do not generally span a holistic process from knowledge co-creation to knowledge application and implementation within one project. Population health studies may have greater health impact when KT is incorporated early and explicitly into the research design. This, we argue, will require that particular attention be paid to collaborative approaches, stakeholder identification and engagement, the nature and sources of evidence used, and the role of the research team working with the local study community.

## Background

### Introduction

Policy and research professionals are actively pursuing ways to move research findings into broader use by communities and frontline staff. Much of this impetus is being led by governmental organizations worldwide [1-3]. In Australia, a government review (McKeon Report) found little connection between health and medical research and its translation into the delivery of healthcare services [4]. The need to develop integrated plans around stakeholder involvement at each step of the research process, as opposed to concentrating on moving research findings into practice at ‘end of grant’ discussions [2,5] is an increasing part of the debate. This connectivity between the knowledge producers and the end-users is implied in definitions of KT, such as that used by the Canadian Institutes of Health Research [6]. However, there is less agreement about how it actually happens in the real world of practice improvement or health system redesign [7,8].

While a substantial body of the KT literature discusses the translation of research into policy and practice [9-11], there is growing attention on the translation of research to communities or defined populations to improve health outcomes [12,13]. The need for much closer partnerships with knowledge users from the beginning of the research process is also an important area for investigation [14,15]. In addition, the context in which evidence is both generated and used is important [6,16]. From these challenges, there is a growing need for explicit approaches to facilitate KT practice within research, especially to support researchers less familiar with KT. A further challenge is that there is much less literature on how population health research teams incorporate KT methodology. This is a gap that needs to be addressed. There has been consideration of the application of KT principles in public health [17], but this has not translated widely to population health debates. Kindig has carefully considered the need to differentiate between public health and population health [18]. He defines population health ‘as health outcomes and their distribution in a population. These outcomes are achieved by patterns of health determinants (such as medical care, public health, socioeconomic status, physical environment, individual behaviour, and genetics) over the life course produced by policies and interventions at the individual and population levels’ [18]. Populations may be defined in various ways (*e*.*g*., age, location, organizational or professional affiliation) and are considered in terms of the variables and factors that affect them as a group rather than as individuals [18]. The concern is with the health outcomes of the defined group, distribution of health concerns amongst that population, and the interventions that might be introduced within that population to improve health outcomes. In our study, the population was defined by place (Port Lincoln) and age (adults). A public health approach would place emphasis on the formalized social, community or organizational activities to maintain, promote or improve health that might also pertain to groups outside our defined population [19,20].

Drawing on our own attempts to create and make explicit an integrated KT approach into a large population health study [21], we argue that existing theoretical approaches for KT are not sufficiently detailed to help researchers and end-users co-create better solutions to healthcare challenges based on the best available, contextualized evidence.

This paper structures the debate as a set of arguments or propositions that have shaped our interrogation of the literature and also informed the development of our integrated KT Framework (called the co-creating or co-KT Framework). The four areas of debate are:

1. There is a need for explicit, theory informed approaches to KT within population health studies;

2. Formalised collaborative approaches need to be evident within any KT framework at each step of the research process;

3. The roles of stakeholders and researchers need to be negotiated, structured and formalized; and

4. KT frameworks need to explicitly describe what counts as evidence, how it is developed, and by whom.

The research study that prompted the theoretical exploration and formulation of a KT framework is a National Health and Medical Research Council (NHMRC) funded project, The Physiology of Health Systems: Port Lincoln as a Case Study (the LINKIN Health Study) [21]. Its aim is to investigate how a regionally defined health system (in South Australia) could gather local evidence of health need and service utilization and use this evidence (along with other externally generated evidence such as clinical guidelines) to redesign the health system. The premise was that by starting off the redesign process with locally derived evidence that would inform more traditionally derived interventions, there would be more likelihood of uptake by the local stakeholders.

We could not locate a suitable KT framework and found ourselves having to address gaps in the pragmatic application of KT for two reasons: KT oriented community and population based studies are limited [22]; and although there are numerous strategies to apply KT principles, guidelines as to the suitability to particular contexts were not easy to ascertain [23]. This paper, therefore, describes how we used the KT literature to build a suitable KT framework for the LINKIN study.

### Proposition 1: there is a need for explicit theory-informed KT approaches to population health studies

KT, as a process, works explicitly to maximize the outcomes of knowledge-producing activity framed to respond to a recognized need or problem. Whether the terms used are research utilization, knowledge transfer, knowledge mobilization, or knowledge utilization [9], they signify that purposeful activities have been undertaken to produce knowledge (information, evidence) with the intent that it can be applied with benefit to the real world. KT therefore in its simplest form, could be conceptualized as something that produces content (the knowledge) and that describes a process (the way the knowledge is adopted and applied in practice or policy). Integral to the process is how the ‘gap’ between the new knowledge created and refined by the researchers is recognized and accepted by the ultimate users of that new knowledge.

There is no one KT theory [24], although existing theories or models have shaped and informed our growing understanding of how KT works. Most influential have been theories of innovation [25,26], behaviour change [27-30], planned change [31,32], organizational change [33-37], and changes to work routines [38,39]. These theoretical approaches are shaping the way that KT interventions are being designed and implemented in practice.

Other research teams have adopted more inductive approaches to developing their conceptual frameworks [40-45]. Rather than identifying an underlying theoretical perspective and building a framework from it, these research teams have taken a more empirical approach, observing and testing practice from which hypotheses are then generated and tested. Many of these teams continue to test the elements of the KT content and processes [43,46-50].

This work reinforces the fact that effective KT is not just about the evidence but is also about the individuals and context into which it is introduced. As a consequence of this wider set of issues, more collaborative KT approaches have been explored. These include incorporating participatory action research approaches [13,51] and considering indigenous KT methods [52]. There are other KT teams who take a more structured methodological approach to understanding and applying the KT process [1,53].

### LINKIN study example

The primary assumption of the LINKIN Study was that if service redesign of a health system was to be successful (measured in terms of better health outcomes for the community; less duplication and waste in the service; and better service delivery), then the first step was to generate evidence of health need and service utilization for the whole community.

In developing our KT approach to research within defined populations, we have drawn on ‘engaged scholarship’ [54] as the theoretical base. Our rationale for embracing this philosophical perspective is fourfold: firstly, the approach grounds the research question in the real world; it is a participative form of research for obtaining the different perspectives of key stakeholders in the study of complex problems; it calls for the use of multiple and competing theories to refine and understand what is actually happening in the real world; and, finally, it advocates the use of a range of analytical strategies such as persuasion, argument, and empirical evidence to arrive at the best knowledge for the particular problem being investigated [55].

A theory-informed framework was needed to show the team where we were going with KT practice and how it fitted with a research process. This helped to overcome that fact that while the concepts behind KT were appreciated by the LINKIN Study team, few had any experience in doing KT within a research project. There was limited experience of working with any KT ideas as a set of concrete tools, measures and components of a large research project. This was addressed by forming a sub group to undertake the detailed work of reviewing the literature and refining the methodological approach. The co-KT Framework provided a way of engaging the whole research team with the new KT concepts, as well as creating a structured approach to community engagement and collecting population-wide data on health need and service utilization.

Our challenge was to create a dynamic, ongoing and methodologically rigorous interaction between the study context (the Port Lincoln community) and the research context (the LINKIN team). We developed a working definition of our KT approach:

Co-KT is a framework for actioning the intent of researchers and communities to co-create, refine, implement and evaluate the impact of new knowledge that is sensitive to the context (values, norms, and tacit knowledge) where it is generated and used.

**Figure 1:**
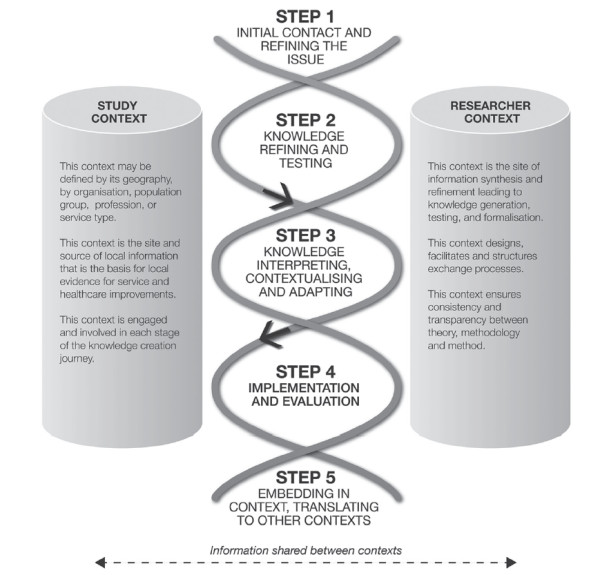
**The co**-**KT Framework.**

Figure 1 summarizes the schema we used to help guide our thinking in the early stages.

The co-KT Framework helped to guide our approach to engaging with the multiple stakeholders (propositions 2 and 3). It also challenged our own implicit assumptions about the nature of local evidence (proposition 4). Table 1 summarizes the steps in the co-KT framework.

**Table 1:** **Steps in the co**-**KT Framework**

***Co-KT Frameworkr***	**Activity**
***Step 1: Initial contact and framing the issue***	Data is conveyed from the study context to the researcher context in response to a query. The initial query may be generated by either context, but will be formally framed by the researcher context.
***Step 2: Refining and testing***	Research team members lead the translation of data and local evidence into a useable commodity by considering existing evidence, the perspectives of multiple stakeholders, and the ongoing input from the study context. This stage may involve conveying information back and forth between the study context and the research context.
***Step 3: Interpreting***, ***contextualising and adapting the knowledge base***	Local evidence is refined and tested against the existing evidence to create intervention ‘prototypes’ to be introduced and tested in the study context.
***Step 4: Implementing and evaluating***	Involvement, trial uptake and response to interventions. Community is engaged and involved in evaluating the impact of the interventions, modifications and considerations for ongoing improvement and sustainability.
***Step 5: Embedding and translating***	Within the study context, new evidence-based interventions are internalized and used to change behaviours, attitudes and work practices. Within the research context, evidence is formalized for local community and for wider scientific community.

### Proposition 2: formalized collaborative approaches need to be evident within any KT framework at each step of the research process

Calls for better research collaboration are not new. What is new is trying to formalize this process within a KT approach that guides the researcher through the translation phases of knowledge creation, refinement, implementation and evaluation, and doing this collaboratively with the range of community stakeholders. Studies that have combined elements of various frameworks to overtly incorporate stakeholder participation suggests that there remain challenges in this undertaking [13].

McWilliam et al., who defined their stakeholders as professionals associated with service provision, acknowledge this complexity [12]. Others have tried to overcome this challenge by the pragmatic adaptation of varied methods as seen in Campbell’s study of a rural community [13]. The approaches Campbell used included the Ottowa Model of Research Use (OMRU) [56], Participatory Action Research (PAR) [3], and Knowledge to Action (KTA) [13]. However, Campbell still found challenges in actually being able to effect change despite the collaborative effort.

In engaged scholarship, researchers and communities, including communities of practice [57] co-create knowledge with a view to improvement of processes and outcomes. The different skills, knowledge and capabilities that researchers and practitioners bring when addressing complex issues as a collaborative venture outweigh either party undertaking the project alone [54]. This is equally applicable to regular community members. This means that the initial product of the KT activity (the knowledge) is generated by both the researchers and the local community participants working together to take the raw material (information) and refining it (through the research and KT process) into a knowledge product (that has the characteristics of credible knowledge, such as veracity, relevance, consistency, ethical base, rigor and logic to it). Here, the emphasis is on working to clarify the stakeholders’ understanding of the knowledge.

We wanted a framework that would allow us to incorporate a variety of engagement strategies that maintained recognition of the distinctive yet complementary roles brought to the study by researchers and the community. This stance was based on our belief that collaboration in the KT process had to be from the beginning knowledge creation stage to the implementation and evaluation stage (see Figure 1). While some frameworks (particularly those from the PAR tradition) reinforce the importance of ongoing collaboration, we could not find any studies that had described the co-creation of knowledge between a population health research team and a local community.

The co-KT Framework also acknowledges the distinct and inevitable separation of the two worldviews of the study (the community and the researcher) context. It does not assume that the community participants will take on the role of researchers, just as researchers cannot become community members. The synergy is in the acknowledgement that the boundaries around the two contexts are real and will persist. The objective is to create constructive and focused dialogue between the boundaries and thereby create a process where information and shared understandings permeate both ways.

The quality of the relationship between the research context and the study (community) context will impact on how knowledge is taken up [58,59]. There also will need to be strategies to bridge the contexts to facilitate information flow, knowledge exchange and knowledge generation [5], and to foster a shared understanding of the value of a planned innovation and its impact [60].

In more traditional KT approaches, it is the research team who identify the evidence and work with a selected group of local stakeholders to consider how best to introduce the new evidence. Collaborative negotiation of need or agreement of the gaps are stages that are not addressed in any great detail, often leading to the target contexts (organizations, health systems, teams of individuals) feeling obliged to participate in a change to which they may have little commitment. We argue that implementation of knowledge is more likely to succeed if the key stakeholders have been involved in the process of knowledge co-creation from the start of the project and are committed to it.

### LINKIN study example

Building on engaged scholarship [55], the LINKIN team considered a range of practical ways of incorporating formal collaboration to address clinical problems that were emerging from the data. For example, from the census data, a major health concern identified was the management of musculoskeletal problems and in particular, the management of pain associated with chronic conditions. Further stakeholder interviews showed that one of the major challenges to patients was streamlining the referral process and ensuring that the multiple health providers (general practitioners, physiotherapists, chiropractors, osteopaths) were able to communicate with each other to deliver an integrated patient care experience. The reality was that few processes had been put in place to enable these groups to talk to each other, so the first task of the research team was to facilitate a series of constructive dialogues with these professional groups and their clients. This was before we could think about introducing any of the evidence-based guidelines on the management of musculoskeletal problems.

### Proposition 3: the roles of stakeholders and researchers need to be negotiated, structured and formalized

The majority of KT literature works on a model of knowledge being generated by experts, and these experts work with potential end users to implement it. Conversely, in frameworks such as PAR, the researchers structure a relationship that supports community members co-leading the research. Neither is sufficient to achieve the full results of effective KT because the research team and local community have distinct and complementary roles to play. These roles cover the phases of identifying, developing and embedding the new knowledge.

In general, strategies for community engagement within KT enquiry may include critical enquiry, community engagement, PAR, interactions between knowledge users and researchers, applications of research products to decision-making processes, and the use of consensus conferences [3,13,61-66]. Most often, this engagement has been described in terms of its impact on knowledge diffusion and uptake, rather than how engagement impacted on the creation of knowledge.

The closest example to what we were trying to do is an interactive KT strategy described by Vingilis et al. [67]. They highlight the importance of involving knowledge users before the end of grant stage so that the research itself might be better informed. This group of researchers integrated knowledge generation with knowledge diffusion and utilization. The research question was generated from within the study context, in response to frustrations put forward by local professionals. The structure was a partnership culture model in which researchers and potential knowledge users worked together to enhance successful knowledge dissemination and utilization. They approached research as the means and not as the end, linking the university and research services to the community, using a participatory research approach. The stakeholders in Vingilis et al.’s study were primarily service providers rather than community members or patients, and the process of developing interventions responsive to an agreed priority was not specified. Vingilis et al. confirmed the importance of having mechanisms where stakeholders and researchers know how to interact with each other.

### LINKIN study example

What was important for the LINKIN team was to acknowledge the involvement of the local community in every step of the KT and research cycle. We started off with the view that by engaging local community personnel in the census collection process, we would have ‘ticked the box’ in terms of community engagement. There were practical structural features in collecting the data, and then there needed to be concerted effort in maintaining the relationship. In setting this up, we used a number of techniques such as the use of research team members who were also local practitioners in the community who could operate as ‘local boundary spanners’ [68]. We also recruited local non-indigenous and indigenous data collectors in gathering quantitative data from households. But we found, having recruited local champions as our ‘boundary spanners,’ that we had entered into a dialogue and relationship with the local community that was beyond pure data collection processes. The community, as exemplified in the action, behaviours and beliefs of the people who were working on behalf of the LINKIN project, believed that this relationship would be able to change and improve services. The research team had to acknowledge that in addition to the requirement to produce high quality research evidence, we also had to work out how we were going to sustain a high quality relationship with our local stakeholders. Issues of time, partnership, resources and commitment arose in these discussions – importantly so as they are the basis for the necessary conversations about embedding changes in any system.

### Proposition 4: KT frameworks need to describe what counts as evidence, how is it developed and by whom

Within KT frameworks, evidence is commonly conceptualized as expertly derived knowledge, refined from a clearly articulated research process and packaged in a way that makes it acceptable to the potential end user [69].

The challenge for the KT researcher is to ensure that the evidence is transferred to the end user in a way that maintains the integrity of the content. Much research effort has been put into understanding how to maintain the fidelity of the evidence content of the intervention and at the same time enable local adaptations and interpretations to be undertaken to optimise uptake [32].

In contrast, and consistent with the more participatory research approaches, there is a growing discourse around how evidence needs to be generated by multiple stakeholders and refined through clear processes of debate and dialogue. Carlile [56] argued that there were three types of knowledge (evidence), each of which could be identified by the way the knowledge reacts to being shifted from one context to another. The first type of knowledge was universally transferrable and moved from one location to another without being changed. It had a common lexicon, or vocabulary, and differences between the ‘new’ and the ‘old’ knowledge were clear. This type of knowledge was codified and technical, and could be ‘transferred’ to another context without change [56].

The second type of knowledge required interpretation and contextualization by those who were to receive it. This process he called translation, because literally, receiving teams or contexts had to interpret the new knowledge and work out ways of making sense of it and adopting it to their own context. The third type was knowledge that, by its nature, was going to challenge the political and power relations in the context receiving the new knowledge. For this (contested) knowledge, the challenge was to engage local stakeholders in a transformation process whereby the social and political structures were engaged in considering the new knowledge [7].

Knowledge (evidence) is not value free. If it is, then it is a type of knowledge that is codified or technical rather than knowledge that requires interpretation and negotiation. Greenhalgh and Wieringa (2011) argue that because of these factors and a number of other challenges in the way KT knowledge production and implementation are conceptualized, it is time to embrace a more integrated notion of evidence and how multiple stakeholders are involved in the process [70]. They also cite Van de Ven’s engaged scholarship [53] approach, suggesting that it is through partnership, mutual respect and ongoing dialogue between the ‘academy’ and the ‘local community’ that shared understandings of knowledge in the form of evidence are arrived at and then actioned.

Van de Ven describes the scientific method as both a way of structuring and analyzing problems to obtain empirical evidence, as well being able to mount sound and persuasive arguments to defend scientific claims. The ability to be persuasive in an argument is not just about the strength of the evidence, but it is also about how the argument is put together (logos); how the emotions are engaged (pathos), and how moral integrity is acknowledged and maintained (ethos) (Van de Ven p65) [26]. Here he would be focusing on the non-codified knowledge that requires the receivers to interpret and make sense of it in their own context (ethical, social and political). Other commentators have raised challenges as to what counts as knowledge or evidence [71].

### LINKIN study example

The LINKIN team wanted to be explicit about how we were going to involve the study community in generating and refining evidence and also in selecting the most appropriate evidence-based interventions for improving services.

We started off by agreeing as a team that the local community would be involved in both the collection of the health census data as well as in the interpretation and validation of it. We fed back preliminary results in accessible, jargon-free ways and ran a series of focus groups with community and health professional stakeholders to get their views on the data. The first area we sought feedback on was the management of musculoskeletal problems. From these conversations, we created more information that would inform the emerging empirical evidence as well as give us a deeper understanding of the wider socio-political and policy issues.

However, there was confusion between the team that was charged with undertaking a systematic review of the evidence around musculoskeletal interventions and how the KT team was going to engage key stakeholder groups (*e*.*g*., those suffering from chronic pain; those who identified problems of early referrals and interprofessional working) in selecting the most acceptable interventions. A set of criteria was developed that enabled both teams to integrate the expert-derived evidence with local stakeholders’ views about what was important and what would be most beneficial to the community.

## Discussion

The propositions that have shaped the arguments in this paper and have informed the development of the co-KT Framework are not new to the KT research community. What is novel is that we have tried to create a theory-informed KT model that would help us manage the tensions between scientific rigour and community engagement; expertly (or externally) derived evidence and locally generated evidence; the nature of the collaborative relationship between the research team and the study community; and how we would prepare the study community for the important intervention or implementation stage.

By using the four propositions as scaffolding upon which to hang our own notions of KT research, we were able to develop a shared understanding of how we would approach KT within a population health study. The co-KT Framework sets out in a series of logical steps the different stages in the overall KT process within the research design. These steps are sequential – tailored interventions cannot be developed without the localized evidence and consultation. However, we also acknowledge that in the actual intervention stage, implementation processes are more characterised by chaotic, non-linear activity [45].

The constant movement between the world of the researchers and the study context reinforced the need for us to be both skilled in the rigour of the scientific method as well as to be able to engage the community in persuasive arguments about the benefits of the work, how we would commit to working with them, and what the likely benefits would be. It soon became clear that the engaged scholarship model combining evidence with persuasive argumentation undertaken in a collaborative, respectful way had a practical validity.

The key steps in the co-KT Framework (See Figure 1) are: knowledge co-creation (from initial contact to transforming local information to evidence); knowledge refinement (which involves the testing and refining of local evidence against existing or expertly derived evidence); knowledge implementation (incorporating interpretation and contextualization and subsequent adoption of the new evidence or knowledge in the local context); and finally the evaluation and refinement stage, including a detailed understanding of what is required to sustain and embed the evidence-based interventions. Embedded within each step are ways of engaging the study community to collaboratively co-create the evolving evidence, validate it and then work on prioritizing which health need will be tackled first in the intervention step.

We anticipate that the strategies for the implementation of the co-KT Framework feature methods for building and sustaining relationships over a long period in anticipation of service redesign proposals or change based on the research evidence. This has implications on the commitment made by research teams to a community, and reflects a number of the elements of the engaged scholarship model [54].

It is important to say a few words about the challenges presented with the conceptual nature of this KT approach. One of the challenges facing us was that the research team reflected the transdisciplinary perspective advocated by Nowotny et al. [72]. This proved to be a challenge in approaching the original research study and in thinking about the wider theoretical issues and the co-KT Framework. For some researchers, engaging stakeholders at each step of the data-gathering process was questioned as to its value. It created complexity, and the cost of holding iterative discussions with stakeholders had to be considered. Other practical challenges that go with such a broad and inclusive KT approach include managing community expectations; managing the sheer volume of process data; managing timeframes; identifying priorities; and facilitating multiple perspectives. These are partly the reasons we chose the engaged scholarship model as a theoretical base for the co-KT Framework. The research activity becomes more of a commitment to an ongoing relationship where researchers and the community are partners in problem-solving and knowledge generation pursuits.

### Summary

The paper proposes the need for an explicit, theoretically informed approach that allows for the incorporation of a collaborative KT process within a population health research study. The KT process includes knowledge generation with stakeholders, knowledge refinement and transfer and knowledge utilization all in one unifying framework. The need for such an approach is illustrated by identifying and addressing four core propositions as they informed and shaped our own experiences in a population health study.

We identify the benefits and challenges of delineating between the researcher and study context roles in the knowledge creating process. These roles are collaborative but different in ways that are productive to all stages of the research process. The researchers orchestrate the process of how data is transformed into knowledge through an iterative, reflective process. It is argued that involving community stakeholders in the problem identification and knowledge creation steps will facilitate better understanding and acceptance of the interventions that are developed ‘in context’ to improve services. The LINKIN project, together with comments and responses to this approach, will help to refine and test the constructs of the co-KT Framework and its working application.

## Competing interests

The authors declare that they have no competing interests.

## Authors’ contributions

ALK led the conceptual development of the co-KT Framework and is responsible for the KT activity within the LINKIN project. She co-wrote and revised drafts of the paper with KP. KP worked on the development of the co-KT Framework, undertook the literature review, co-wrote, refined and led the editing of multiple drafts of the paper. She is responsible for implementing the co-KT strategy within the LINKIN project. EH worked with KP on refining the co-KT Framework, commented on drafts and is project lead for LINKIN. JN commented on drafts and is one of the local boundary spanners in Port Lincoln. AW commented on drafts and has facilitated stakeholder meetings with KP and EH in Port Lincoln. JB leads the conceptual and research design of the LINKIN study and has reviewed all drafts. All authors read and approved the final manuscript.
